# Adherence to the oral contraceptive pill: a cross-sectional survey of modifiable behavioural determinants

**DOI:** 10.1186/1471-2458-12-838

**Published:** 2012-10-02

**Authors:** Gerard J Molloy, Heather Graham, Hannah McGuinness

**Affiliations:** 1 Division of Psychology, School of Natural Sciences, University of Stirling, Stirling, Scotland, UK; 2 School of Psychology, National University of Ireland Galway, Galway, Ireland

**Keywords:** Adherence, Compliance, Oral contraception pill, Intention, Anticipated regret, Beliefs, Planning, Behaviour

## Abstract

**Background:**

Poor adherence to the oral contraceptive pill (OCP) is reported as one of the main causes of unintended pregnancy in women that rely on this form of contraception. This study aims to estimate the associations between a range of well-established modifiable psychological factors and adherence to OCP.

**Method:**

A cross-sectional survey of 130 female University students currently using OCP (Mean age: 20.46 SD: 3.01, range 17–36) was conducted. An OCP specific Medication Adherence Report Scale was used to assess non-adherence. Psychological predictor measures included necessity and concern beliefs about OCP, intentions, perceived behavioural control (pbc), anticipated regret and action and coping planning. Multiple linear regression was used to analyse the data.

**Results:**

Fifty-two per cent of participants reported missing their OCP once or more per month and 14% twice or more per month. In bivariate analysis intentions (r = −0.25), perceived behavioural control (r= −0.66), anticipated regret (r=0.20), concerns about OCP (r =0.31), and action (r= −0.25) and coping (r= −0.28) planning were all significantly associated with adherence to OCP in the predicted direction. In a multivariate model almost half (48%) of the variation in OCP adherence could be explained. The strongest and only statistically significant predictors in this model were perceived behavioural control (β=−0.62, p<0.01) and coping planning (β =−0.23, p=0.03). A significant interaction between intentions and anticipated regret was also observed.

**Conclusion:**

The present data point to a number of key modifiable psychological determinants of OCP use. Future work will establish whether changing these variables results in better adherence to the OCP.

## Background

The oral contraceptive pill (OCP) remains the most popular form of contraception among young women in the UK with 54% of women who use contraception between the age of 20–24 years using the OCP [1]. The success of this method is however highly dependent on good adherence to the prescribed OCP daily regimen. International studies suggest that up to 47% of women do not fully adhere to OCP with 22% missing two or more OCPs per cycle [2]. Missing or forgetting to take the OCP is reported as one of the main reasons for seeking emergency contraception [1] in women that rely on OCP for contraception. Poor adherence to OCP may therefore be a primary cause of unintended pregnancy in those that rely on this method of contraception, which is an important concern for public health [3].

There are a number of socio-demographic and clinical variables that have been linked to poor adherence to OCP, such as lower level of education, having a lower income, having an occasional partner and experiencing side effects from OCP use [4]. Psychological approaches are useful in attempts to understand adherence to any kind of medication regimen in that they can identify potentially modifiable variables [5] such as beliefs about the necessity of medication or specific concerns about medications [6,7] or the strength of intentions to use medication and confidence about using medications [8] that may be causally related to medication taking [9]. This literature also specifies a range of behaviour change techniques [10] that can be applied to problems such as non-adherence. Therefore use of the OCP may be better understood by examining the links between these established psychological predictors and measures of adherence to OCP.

Psychological theories of health behaviour have developed in recent years to encompass a broader range of cognitive determinants of behaviour than set out in early theoretical formulations [11]. For example it has been proposed that ‘anticipated regret’, a negative cognitive based emotion where we anticipate a negative emotion to result from a particular behavioural choice, should be added to social cognitive theories of behaviour [12] as it may be a key moderator of the relationship between an individual’s intentions to engage in a behaviour and their actual behaviour. Another related body of work focuses on the processes that follow forming an intention to engage in a behaviour such as making action or coping plans [13]. These planning processes are thought to reduce the influence of unwanted automatic behaviours and to enhance the formation of habits i.e. context dependent responses acquired through repetition. These developments in theory and the related measurements have not been widely considered in relation to OCP adherence.

In this study we therefore examine whether seven key potentially modifiable psychological variables from a number of influential psychological approaches to health behaviour are associated with OCP adherence. We hypothesise that better adherence will be associated with lower concerns about the OCP, higher necessity beliefs relating to the OCP, higher intentions to use OCP, higher perceived behavioural control over using OCP, higher anticipated regret about not using the OCP and higher action and coping planning in relation to OCP.

## Methods

### Design

In this cross-sectional study data were collected from female undergraduate students at a University setting between October and December 2011.

### Participants and procedure

Informed consent was provided by 130 students. The mean age of the sample 20.46 years with a standard deviation of 3.01 years and an age range 17–36 years. Ninety-one per cent of participants were ≤ 22 years old. A web-based questionnaire was added to an online experiment management system and female students who were currently using OCP were invited to take part. Ethical approval for the study was granted by the university psychology ethics committee.

### Measures

#### Adherence to OCP

The medication adherence report scale (MARS) was used to measure adherence to OCP as a continuous variable [14]. The five items used in this study were modified slightly to indicate that they referred to OCP use. Adapting the MARS in this way has been shown to be acceptable in other clinical groups [15]. The five items used were as follows: I forget to take my oral contraceptive pill; I alter the dose of my oral contraceptive pill; I stop taking my oral contraceptive pill for a while when I am not supposed to; I decide to miss out a dose of my oral contraceptive pill; I take less than instructed. Responses to these items were on a 5 point scale from 1 Never to 5 Always. Higher scores related to poorer adherence. Participants were also asked, ‘How many times on average, do you miss your oral contraceptive pill each month?’ with three options for responses of 0, 1 or 2+ times per month.

#### Theory of planned behaviour and anticipated regret

The proximal predictors of behaviour from the theory of planned behaviour were measured using items adapted from existing scales used in other behaviours [16]. The intention items were: ‘I expect to take my pill as prescribed’ and ‘I want to take my pill as prescribed’. The perceived behavioural control (PBC) items were: ‘To what extent do you see yourself as being capable of taking your pill as prescribed?, ‘How confident are you that you will be able to take your pill as prescribed?’, ‘I believe that I have the ability to take my pill as prescribed’. Both were measured with a 5 point response scales e.g. from 1 Strongly disagree– 5 Strongly agree. Higher scores related to stronger intentions and stronger perceived behavioural control. Anticipated regret was measured using two items based on existing scales [12]. The two items were: ‘If I did not take my pill as prescribed, I would feel regret’ and ‘If I did not take my pill as prescribed, I would feel upset’. Responses to these items were on a 5 point scale from 1 Definitely Yes to 5 Definitely no. Higher scores related to lower anticipated regret.

#### Beliefs about medications

Necessity and Concerns beliefs from the Necessity-Concerns framework [17] were assessed using 8 items adapted from Horne and Weinman’s Beliefs about Medications Questionnaire-specific [14]. The items were modified to refer to OCP use. The three Necessity items were, ‘My health at present depends on my oral contraceptive pill’, ‘My life would be impossible without my oral contraceptive pill’ and ‘My health in future will depend on my oral contraceptive pill’. The Concerns items were, ‘Having to take oral contraceptives worries me’, ‘I sometimes worry about the long-term effects of my oral contraceptive pill’, ‘My oral contraceptive pill is a mystery to me’, ‘My oral contraceptive pill disrupts my life’ and ‘I sometimes worry about becoming too dependent on my oral contraceptive pill’. Responses to these 8 items were on a 5 point scale from 1 Strongly disagree–5 Strongly agree. Higher scores related to stronger Necessity beliefs and greater Concerns about OCP use.

#### Planning

Action planning was measured using a four-item scale based on a widely used measure [18]. Items began with the stem ‘I have made a detailed plan regarding…., when to take my oral contraceptive, where to take my oral contraceptive, how to take my oral contraceptive, how often to take my oral contraceptive’. The responses to the five items were on a five-point scale 1 Strongly disagree–5 Strongly agree. Coping planning was measured using a five-item scale also based on the same existing measure. Items also began with the stem ‘I have made a detailed plan regarding…, what to do if something interferes with my plans for taking my oral contraceptive, how to cope with possible setbacks with my plans for taking my oral contraceptive, what to do in difficult situations in order to act according to my intentions for taking my oral contraceptive, good opportunities for taking my oral contraceptive, when I have to pay extra attention to prevent lapses from taking my oral contraceptive’. The responses to the five items were on a five-point scale 1 Strongly disagree–5 Strongly agree. Higher scores related to stronger formation of action and coping plans for OCP use.

#### Other socio-demographic and background factors

The questionnaire also recorded information on age, relationship status, whether participants were currently sexually active, their duration of OCP use, whether they used a 21 day or 28 day OCP, whether they had ever used emergency contraception, whether they experienced side effects and whether they had a fixed e.g. morning, afternoon, evening or bedtime or variable time for their OCP use. This was a dichotomous variable i.e. fixed versus variable.

#### Analysis

Descriptive statistics were used to examine means, variability and bivariate correlations between the main study measures. Parametric and non-parametric tests where appropriate were applied to compare groups of participants. Internal consistency for multi-item scales was calculated using Cronbach’s alpha. As the dependent variable, Adherence to OCP, was positively skewed this was square root transformed. This reduced skewness and the transformed variable was used for all analysis. In order to assess the predictive power of the 7 psychological variables a hierarchical multiple regression analysis was conducted with Intention, perceived behavioural control and regret entered in the first step, necessity and concern beliefs about OCP entered in the 2^nd^ step and finally in the third step action and coping planning were entered. This approach was taken to establish the incremental validity of each set of predictors beyond an initial theory of planned behaviour and anticipated regret motivational model. Mullticollinearity was assessed by examining the variance inflation factor (VIF). All P values in statistical tests refer to two tailed tests. Post-hoc power analysis revealed that the recruited sample size of 130 and the lowest sample size (N=117, due to missing data on one or more measures) used in multivariate analysis had over 80% power to detect medium effect sizes (f^2^= 0.15) in a multiple regression model with 7 tested predictors and an alpha level of 0.05. We also separately tested in a supplementary analysis whether there was an interaction between intention to use OCP and anticipated regret as has been observed in other health behaviours using moderated regression analysis [12,19], as the study sample size had limited power to include interaction terms in models with multiple predictors.

## Results

Table 1 presents descriptive statistics for the key study measures by the three subgroups that emerged from the question ‘How many times, on average, do you miss your oral contraceptive pill each month?’ There were no significant differences between the 3 groups in background and demographic factors apart from on the question asking whether participants had a fixed time for OCP use. Participants who didn’t have a fixed time for OCP use were much more likely to miss their OCP once or more (Odds ratio 3.62, 95% confidence interval: 1.34-9.79). The correlation between the continuous (MARS) and categorical measures (0, 1 or 2+ times per month) of Adherence to OCP was positive and large (r = 0.57, p<0.01) providing evidence of convergent validity for the two measures.

**Table 1:** Study sample by ‘How many times, on average, do you miss your oral contraceptive pill each month?’

	**Never**	**Once**	**Two or more times**	**P**
	**(N=62, 48%)**	**(N=50, 39%)**	**(N=18, 14%)**	
Age - Mean (SDs)	20.77 (3.37)	20.18 (3.01)	20.17 (1.10)	0.53
Single relationship status - N (%)	23 (37)	24 (48)	5 (28)	0.26
Currently sexually active - N (%)	54 (87)	37 (76)	17 (94)	0.11
Using OCP less than 1 year - N (%)	16 (26)	12 (25)	7 (39)	0.48
OCP 21 day- N (%)	50 (81)	40 (82)	13 (77)	0.90
Used emergency contraception - N (%)	31 (51)	17 (34)	11 (61)	0.08
Side effects experienced - N (%)	23 (37)	18 (37)	6 (33)	0.96
Fixed time for OCP use - N (%)	56 (90)	41 (82)	8 (44)	<0.01
		Mean (SDs)		
Intention	4.65 (0.51)	4.55 (0.53)	4.28 (0.43)	0.03
Perceived behavioural control	4.76 (0.40)	4.49 (0.52)	3.59 (0.84)	<0.01
Anticipated regret	2.16 (1.16)	2.47 (0.91)	2.69 (0.91)	0.10
Necessity beliefs	2.18 (0.69)	2.16 (0.70)	2.13 (0.44)	0.96
Concern beliefs	2.25 (0.68)	2.45 (0.60)	2.63 (0.55)	0.05
Action planning	3.47 (1.03)	3.11 (0.87)	2.63 (0.99)	<0.01
Coping planning	3.71 (0.92)	3.26 (0.76)	2.99 (1.01)	<0.01

With regard to the psychological predictors there were significant differences between the three groups on all measures with the exception of anticipated regret and necessity beliefs. The significant differences observed were all in the predicted direction with those reporting never missing their oral contraception each month with higher intentions to use OCP, higher perceived behavioural control, lower concerns about OCP and higher levels of action and coping planning.

Table 2 presents the correlation matrix together with the means and standard deviations and the Cronbach’s alphas for the main study measures. All of the psychological predictors with the exception of Necessity beliefs were significantly associated with adherence to OCP in the predicted direction. Effect sizes were mostly in the small to medium range (r >0.10 and r< 0.30), according to Cohen’s criteria [20], with the exception of PBC which had a large association with OCP adherence i.e. r > 0.50. The Cronbach’s alpha for all scales was satisfactory (alpha > 0.70) with the exception of Necessity beliefs.

**Table 2:** Descriptive statistics and correlation matrix for the main study measures

	**1.**	**2.**	**3.**	**4.**	**5.**	**6.**	**7.**	**Mean (SD)**	**Cronbach’s alpha**
1. Adherence to OCP	1							2.71 (0.40)	0.73
2. Intention	-.33^**^	1						4.56 (0.52)	0.82
3. PBC	-.62^**^	.39^**^	1					4.49 (0.65)	0.91
4. Anticipated regret	.20^*^	-.10	-.04	1				2.35 (1.05)	0.85
5. Necessity beliefs	.08	-.19^*^	-.14	-.03	1			2.16 (0.66)	0.57
6. Concern beliefs	.29^**^	-.25^**^	-.38^**^	.01	.50^**^	1		2.38 (0.65)	0.70
7. Action planning	-.24^**^	.28^**^	.23^*^	-.21^*^	.09	-.03	1	3.22 (0.99)	0.87
8. Coping planning	-.26^**^	.36^**^	.19^*^	-.13	-.09	-.09	.73^**^	3.44 (0.91)	0.93

**p< 0.01; *p< 0.05.

Table 3 presents the hierarchical multiple regression of Adherence to OCP on the seven psychological predictors. Although action and coping planning were highly correlated multicollinearity was not a problem in this analysis according to the VIF values. The final model accounted for almost half of the variation in adherence to OCP. Although the final model in Step 3 did account for an additional 4% of the variability beyond that accounted for the initial model with Intention, PBC and regret, there was not a statistically significant change to the R^2^ in steps 2 (p=0.57) and step 3 (p=0.06). The strongest and only statistically significant predictors in the final model were perceived behavioural control and coping planning. Higher levels of perceived behavioural control and higher levels of coping planning were associated with better adherence to OCP. We re-ran the analysis with the problematic Necessity variable excluded and this did not significantly alter the overall pattern of findings. We also ran this model using unintentional (Item 1: I forget to take my oral contraceptive pill) and intentional MARS items (other 4 items) as dependent variables in a sensitivity analysis. The R^2^ for unintentional non-adherence was 0.46, p<0.001 while the R^2^ for intentional non-adherence was 0.35, p<0.001, with pbc and coping planning remaining as the strongest predictors in both models.

**Table 3:** Hierarchical multiple regression analysis of Adherence to OCP on psychological predictors

	**R**^**2**^	**ΔR**^**2**^	**B**	**β**	**F change**	**Lower 95% CI B**	**Upper 95% CI B**
	0.44**	0.44**			29.83		
Step 1							
Intention			0.001	0.002		−0.114	0.116
PBC			−0.374	−0.651		−0.460	−0.287
Anticipated regret			0.040	0.110		−0.011	0.091
Step 2	0.45**	0.01			0.57		
Intention			0.004	0.005		−0.113	0.121
PBC			−0.357	−0.622		−0.450	−0.265
Anticipated regret			0.040	0.111		−0.011	0.091
Necessity beliefs			−0.030	−0.053		−0.124	0.063
Concern beliefs			0.055	0.094		−0.049	0.160
Step 3	0.48**	0.03			2.97		
Intention			0.047	0.061		−0.074	0.167
PBC			−0.354	−0.617		−0.446	−0.262
Anticipated regret			0.038	0.104		−0.013	0.089
Necessity			−0.045	−0.078		−0.140	0.051
Concerns			0.065	0.109		−0.038	0.168
Action planning			0.032	0.084		−0.049	0.113
Coping planning			−0.097	−0.232		−0.186	−0.008

**p< 0.01; *p< 0.05; 2206;R^2^= R^2^ change; B =Beta; β = Standardized Beta.

**Figure 1:**
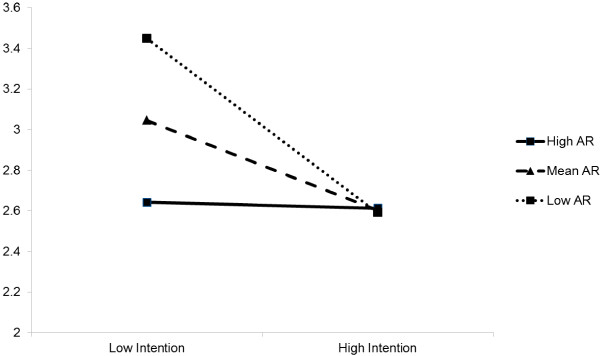
Interaction between anticipated regret (AR) and intention on OCP non-adherence (higher scores on the y axis relate to more non-adherence to OCP).

A simple moderated regression analysis revealed an interaction between intentions and anticipated regret. The R^2^ increase due to the interaction was 0.09, p<0.001 and the beta for the interaction term was - 0.207, standard error = 0.056, p<0.001. The probed interaction is illustrated in Figure 1. We conducted slopes analysis on the low and high anticipated regret lines to determine whether they differed from zero. This revealed that the line for low anticipated regret differed significantly from zero (Beta =−0.438, standard error= 0.081, p<0.001), but the line for high anticipated regret did not (Beta =−0.006, standard error =0.088, p =0.948).This shows that intention to use OCP and adherence to the OCP relationship was moderated by anticipated regret. In other words intentions to use the OCP were strongly predictive of non-adherence when anticipated regret was low but not when regret anticipated regret was high.

## Discussion

The observation in this study that approximately half of the sample reported some level of non-adherence to their OCP is consistent with previous international studies [2]. The data demonstrated an association between six of the seven psychological predictors and adherence to OCP. All of the psychological variables included in the present study are well-established in the health behaviour literature and are derived from clearly articulated psychological theory relating to the self-regulation of health related behaviour [5,12-14]. The multivariate model shows that perceived behavioural control and coping planning were the most powerful predictors of adherence to OCP in this sample. The detected interaction between intention and anticipated regret reveals that some of these variables may have synergistic non-linear effects in their relationship with adherence to OCP. In this instance for example lower anticipated regret in combination with lower intentions was particularly predictive of poor adherence to OCP. All the variables considered in the main analysis are potentially modifiable [10] and may therefore represent important targets for change in any complex interventions that aim to enhance adherence to OCP.

Both intention and perceived behavioural control have been linked to medication adherence in patients receiving immunosuppressant therapy and the present findings are consistent with these data [8].The findings in this study are also consistent with studies that have looked at medication beliefs in those with chronic and acute illness [6,21], which have identified associations of comparable size between medication concerns and measures of adherence. The relatively small association between necessity beliefs relating to OCP and adherence observed in this study may be due to the availability of other forms of effective contraception e.g. condoms and emergency contraceptive pill, which can provide the same ultimate effect of OCP. It is also possible that the study measure of necessity beliefs did not precisely capture this construct in the context of OCP use, as our Cronbach’s alpha value was lower than conventional thresholds i.e. alpha > 0.70. Therefore an improved measure of necessity beliefs is required before this approach [17] can be properly tested. For example, a revised scale could begin with the stem “Avoiding unwanted pregnancy” instead of “My life/future health”. This may result in a more internally consistent scale and would improve the face validity of the measure.

We are not aware of any studies that examined the link between action and coping planning and medication adherence nor are we aware of any studies that have looked at the association between anticipated regret and medication adherence. Therefore these new observations are not limited to the context of OCP use, but are new findings in terms of medication adherence literature more generally.

### Strengths and limitations

This is the first study to examine a wide range of psychological predictors of adherence to OCP and provide new information that can inform future studies aiming to understand and change adherence to OCP. There are however a number of limitations to the methodology which should be considered when interpreting the results. First, the cross-sectional correlational design cannot be equated with causality in any way. The limitations of this kind of design in the domain of health behaviour have been clearly articulated [22], however as this is the first study to examine some of the relationships between these variables, this limitation should be considered in terms of the initial stages of inquiry into this specific area. Second, the measurement of adherence could have been improved by using multiple methods, particularly more objective methods [23]. Serum or urine assays [24,25] for example provide direct evidence of adherence to OCP, however these methods can be expensive and difficult to collect therefore self-report remains the mainstay in this area of research because of these challenges [23]. It is important to note that some problems with self-report methodology such as ‘social desirability’ biases that can influence data collection in face-to-face interview studies [23] were likely to be reduced in this study, as data was collected through an anonymous web based questionnaire and participants did not provide any personal identifiers. Finally the sample was likely to be restricted in terms of socioeconomic status and the level of education of the participants was clearly on average higher than the population in general, as participants were recruited from a University setting. This did not however result in a very limited range in the distribution of our measure of non-adherence to OCP, which is likely to be a problem for all socio-economic groups and education levels, albeit to a much greater extent in lower socio-economic groups [4]. Levels of non-adherence to OCP in this study were also comparable to larger international studies with more diverse samples [2].

### Future work

Replication of these findings in longitudinal study designs is now required to establish whether changes in these predictors over time leads to changes in adherence to OCP over time. Ideally future studies would use more than one method of OCP adherence and in particular objective direct indicators which could validate self-report findings [23]. This work would provide a stronger foundation for experimental studies that test specific interventions that aim to enhance adherence to OCP. Experimental studies are necessary to identify the key causal psychological variables in adherence to OCP [22].

### Implications

From a public health perspective these findings are important in that they confirm that over half of young women using the OCP may be failing to fully adhere to the regimen. As unplanned pregnancies can pose significant health and social difficulties for the individual and society [3], reliable and effective methods of enhancing adherence to the current contraceptive of choice among young women i.e. the OCP are required. Available reviews of interventions indicate that evidence in this area is limited [26]. The present findings are also important in that they clearly underline the importance of pill user confidence and planning in relation to using the OCP. The observation that 90% of those that never miss their OCP take it at the same time every day versus only 44% of those that miss it twice or more a month clearly demonstrate that forming a time related habit may be critical to the successful use of the OCP. Taking the OCP at the same time each day can also influence contraceptive efficacy. Therefore there may be a benefit from simply encouraging users to make specific plans about when, where and how to take their OCP i.e. making an action plan, and what to do when faced with obstacles to taking OCP i.e. making a coping plan. A recent randomised controlled trial in the UK has provided evidence that a simple planning intervention where young women specified when, where and how to obtain or use their preferred contraceptive method and how to overcome barriers to carrying out these plans with pencil and paper was effective in reducing emergency contraception provision or pregnancy testing over a 2 year period [27-29]. The results of this trial provide initial evidence that simple behaviour change techniques that could be applied in seconds in a clinical setting can have a significant enduring impact on changing contraception related behaviour in adolescents and young women. Some commentators have recommended adopting these approaches into standard practice in family planning settings while further confirmatory research is conducted [30].

## Conclusion

The present data suggests that behaviour change strategies that can enhance PBC and coping planning for OCP use may lead to improved adherence. This could reduce risk for emergency contraception use and unintended pregnancy. Intervention studies are now required to establish whether changing these variables results in better adherence to OCP.

## Abbreviations

OCP: Oral contraceptive pill; PBC: Perceived behavioural contro; MARS: Medication adherence report scale; AR: Anticipated regret; VIF: Variance inflation factor.

## Competing interests

The authors declare that they have no competing interest.

## Authors’ contributions

GJM conceived and designed the study with HG and HMCG. HG and HMCG designed the online questionnaire and collected the data. GJM led the statistical analysis and interpretation of the data and writing the paper. All authors read and approved the final manuscript.

## Pre-publication history

The pre-publication history for this paper can be accessed here:

http://www.biomedcentral.com/1471-2458/12/838/prepub
